# Composition of volatile compounds and in vitro antimicrobial activity of nine *Mentha* spp.

**DOI:** 10.1186/s40064-016-3283-1

**Published:** 2016-09-21

**Authors:** Yun Ji Park, Thanislas Bastin Baskar, Sun Kyung Yeo, Mariadhas Valan Arasu, Naif Abdullah Al-Dhabi, Soon Sung Lim, Sang Un Park

**Affiliations:** 1 Department of Crop Science, Chungnam National University, 99 Daehak-ro, Yuseong-gu, Daejeon, 305-764 Korea; 2 Department of Botany and Microbiology, Addiriyah Chair for Environmental Studies College of Science, King Saud University, P. O. Box 2455, Riyadh, 11451 Saudi Arabia; 3 Department of Food Science and Nutrition and Institute of Natural Medicine, Hallym University, Chuncheon, 200-702 Korea

**Keywords:** *Mentha*, Volatile compounds, Antibacterial activity, GC–MS

## Abstract

**Background:**

*Mentha* plants containing over 25 species are aromatic perennial herbs. These species have been interested and widely used because of various clinical findings. Many volatile compounds facilitate environmental interactions such as protecting themselves from pathogens, parasites, and herbivores. Therefore, this study assessed comparison of volatile composition and antimicrobial activity from nine *Mentha* species. The composition of volatiles was investigated from the aerial parts of nine different *Mentha* species using gas chromatography-mass spectrometry (GC/MS). In addition, screened antimicrobial activities against six food borne pathogenic bacteria using extracts obtained these plants.

**Results:**

77 volatile compounds were identified in total and it included 13 monoterpenoids, 19 sesquiterpenoids, and others. In particular, monoterpenoids such as eucalyptol (9.35–62.16 %), (±)camphorquinone (1.50–51.61 %), and menthol (0.83–36.91 %) were mostly detected as major constituents in *Mentha* species. The ethanol extract of nine *Mentha* species showed higher activity compared to other solvent extracts (methanol, hexane, di ethyl ether). Among these nine *Mentha* species chocomint showed higher inhibition activity against all bacteria.

**Conclusions:**

It is concluded that monoterpenoids are mainly rich in *Mentha* plants. Moreover, most of extracts obtained from *Mentha* showed strong antimicrobial activity against bacteria. Of these, chocomint indicates the highest inhibition activity.

## Background

Mint (*Mentha* spp.), a genus of aromatic perennial herbs, is included in the family Lamiaceae. The genus *Mentha* comprises more than 25 species which found mostly in temperate and sub-temperate areas of the world (Bhat et al. 2002). Among the *Mentha* species, peppermint, spearmint, wild mint, curled mint, American mint, bergamot, Korean mint are common (Shaikh et al. 2014). Since ancient times mint is popular and widely used in cuisines, medicines, and cosmetics due to many benefits for human health (Saeed et al. 2006). For instance, this plant provides relief from common cold, fever, flu, indigestion, and motion sickness (Therdthai and Zhou 1994). Besides, a lot of items of daily use including confectionary, cosmetics, oral hygiene products, pharmaceuticals, pesticides, and as a flavor enhancing agent in toothpastes, chewing gums and beverages contains fresh plants or their essential oils form as ingredients (Eccles 1994; Croteau et al. 2005). Several mint species are distributed all across the globe for cultivation as industrial crops (Bhat et al. 2002). According to the fact, *Mentha* plays an important role economically. Numerous researches have been investigated to isolate and distinguish the constituents including flavonoids, phenolic acids, terpenoids, and other volatile compounds from various extracts of *Mentha* species. This plant with various therapeutic values such as antidiarrheal, cardiovascular, and central nervous system (CNS) effects, and antimicrobial, antioxidant, and anti-inflammatory activities has the hopeful potential as a medicinal herb (Shaikh et al. 2014).

Volatile organic compounds, commonly lipophilic liquids with high vapor pressures, represent the largest group of natural products in plants. These compounds have multiple effects on both floral and vegetative tissues (Pichersky et al. 2006). Generally, many floral volatiles serve to attract pollinators and also act as protectors for valuable reproductive parts of plants against pathogens, parasites, and herbivores (Dudareva et al. 2004). Vegetative volatiles involve in signaling of inter-plant or inner plant organs and plant defense against pathogens, heat, and oxidative stress (Unsicker et al. 2009). Floral scents attracting pollinator have widely given delight to the human’s olfactory sense for long times. Besides, numerous aromatic plants have been used as flavorings, preservatives, and herbal remedies (Pichersky et al. 2006). The most common constituents of plant volatiles are terpenoids, phenylpropanoids/benzonoids, fatty acid derivatives, and amino derivatives (Dudareva et al. 2013). Terpenoids, as the largest and diverse class of plant secondary metabolites with many volatile constituents, are derived from two basic C5 units, isopentenyl diphosphate (IPP) and dimethylallyl diphosphate (DMAPP) (McGarvey and Croteau 1995).

In recent years, there is an increasing need of drug development because of pathogens resistance to many antibiotics so, many researchers investigate on that new drug development (Balachandran et al. 2015; Santhosh et al. 2016). In last decade, the bacterial diseases highly found in poor population countries because of many bacteria resistance to the antibiotics so, require developing antibacterial compounds (Ahameethunisa and Hopper 2010). Various diseases like cancer, complication of chronic conditions, transplants, and AIDS has been incriminating by the bacterial strains because of low immunity power (Cragg et al. 1997; Panghal et al. 2010). The main source of bioactive compound derived from plant species because of their low toxicity and 77 % of important drugs were derived from the traditional medicinal plants which are used in many diseases (Cragg et al. 1997).Gram-positive and Gram-negative bacteria growth was prevented by peppermint oil and menthol have been demonstrated previously (Quevedo Sarmiento and Ramos Cormenzana 1988). Peppermint is also having antiviral and fungicidal activities were revealed (Chaumont and Senet 1978). Also the essential oil of *M. piperita* is commonly used in folk medicine for respiratory diseases as cough syrup and anti-congestive (Vieira 1992; Ody 2000; Corrêa et al. 2003) and as antispasmodic on the digestive and vascular systems (Ody 2000). Antispasmodic effect of *M. piperita* essential oil on tracheal smooth muscle of rats was already reported (de Sousa et al. 2010). The aim of the present study was to determine the volatiles profile using gas chromatography–mass spectrometry in different nine *Mentha* species including *M. piperita*, *M. pulegium*, *M. spicata*, *M. longifolia*, *M. aquatica*, *M. suaveolens*, and *M x piperita* (two hybrids). Also, we evaluated antimicrobial activity against some pathogenic bacteria using different *Mentha* extracts.

## Results

### Volatile constituents of nine *Mentha* species

The identified volatile constituents in different *Mentha* plants are shown in Table 1. 77 volatile components were found based on comparison of the mass spectrum in total. The content of plant volatiles expressed in percentages was as follows: peppermint (*M. piperita)*, 98.27 %; water mint (*M. aquatic)*, 94.95 %; apple mint (*M. suaveolens)*, 98.54 %; spearmint (*M. spicata)*, 97.42 %; chocolate mint (*M x piperita* ‘Chocolate’), 99.70 %; pineapple mint (*M. suaveolens* ‘Variegata’), 97.02 %; horsemint (*M. longifolia)*, 99.70 %; eau de cologne mint (*M x piperita* f. citrate), 97.84 %; pennyroyal mint (*M. pulegium)*, 99.81 %. The dominant components inpeppermint were eucalyptol (62.16 %), 4-Terpineryl acetate (6.17 %), and menthol (4.30 %). (−)-calamenene (12.17 %), eucalyptol (11.39 %), citronella (11.04 %), and α-gurjunnene (10.88 %) were mainly detected in water mint. (±)camphorquinone (51.61 %), eucalyptol (19.49 %), and γ-terpinene (5.25 %) were obtained as main constituents of volatiles in apple mint. The main constituents in spearmint were eucalyptol (46.28 %), 1,3,5-tri-Methyl-6-methylene-cyclohexene (19.25 %), and caryophyllene (5.47 %). Chocolate mint indicated menthol (36.31 %) as the major constituent, followed by eucalyptol (22.70 %), and D-limonene (7.91 %). Pineapple mint were characterized by a high content of eucalyptol (37.36 %), followed by γ-terpinene (16.94 %), 4-terpineryl acetate (10.53 %). In horsemint, the major compounds were *p*-menthan-3-one (31.24 %), eucalyptol (9.35 %), and 1,3-diethylbenzene (6.02 %). Eau de cologne mint was characterized by the dominant presence of eucalyptol (18.36 %), caryophyllene (9.81 %), and *p*-methan-3-one (8.13 %). Other significant constituents in this species included 1,3-cyclohexandiene (6.40 %), 1-methyl-4-(methylidene)cyclohexane (6.31 %), and 2,4,6-oxtatriene (6.28 %). The most abundant constituents in pennyroyal mint were *p*-methan-3-one (71.87 %) and menthol (11.29 %).

**Table 1:** Volatile component composition (peak area  %) and their amount of nine *Mentha* spp.

Volatile compound	Peak area (%)^a^									
	RT	PP	WT	AP	SP	CC	PA	HS	ED	PR
1,3-Diethylbenzene	7.92	ND	ND	ND	ND	ND	ND	6.02 + 0.59	ND	ND
3,5-Dimethylanisole	8.63	ND	ND	ND	ND	ND	ND	2.50 + 0.22	0.49 + 0.04	ND
1,2,3,4,5-Pentamethy-1,3-cyclopentadiene	8.83	ND	ND	ND	ND	ND	ND	0.22 + 0.02	0.54 + 0.05	ND
1,3,3-Trimethyl-tricyclo[2,2,1,0(2,6)]heptane	9.43	ND	ND	0.05 + 0.01	ND	ND	ND	0.10 + 0.01	ND	ND
γ-Terpinene	9.59	5.09 + 0.49	0.18 + 0.01	5.25 + 0.51	1.34 + 0.11	2.28 + 0.21	16.94 + 1.72	2.15 + 0.19	ND	0.34 + 0.03
3-(1-Phenylethoxy)-butanoic acid	9.7	ND	ND	ND	ND	ND	1.39 + 0.12	0.02 + 0.01	ND	ND
2,7-Dimethyl-(*Z*)-3-octen-5-yne	10.41	ND	0.66 + 0.06	ND	0.02 + 0.01	ND	ND	0.01 + 0.01	1.59 + 0.12	0.21 + 0.02
Terpinolene	10.84	ND	ND	1.40 + 0.12	ND	0.86 + 0.08	ND	1.08 + 0.11	ND	ND
4-Terpineryl acetate	10.95	6.17 + 0.59	1.56 + 0.11	3.72 + 0.31	3.20 + 0.29	2.86 + 0.24	10.53 + 1.03	2.49 + 0.23	ND	0.25 + 0.02
Benzaldehyde	11.25	ND	ND	0.04 + 0.01	0.01 + 0.01	0.04 + 0.01	ND	ND	ND	ND
1,3,5-tri-Methyl-6-methylene-cyclohexene	11.6	0.58 + 0.44	ND	0.07 + 0.01	19.25 + 1.88	0.04 + 0.01	ND	1.01 + 0.10	0.50 + 0.05	0.21 + 0.02
6-Isopropenyl-3-methoxy-3-methyl-cyclohexene	12.01	ND	ND	2.03 + 0.21	ND	ND	ND	0.98 + 0.08	0.75 + 0.07	ND
2-Carene	12.25	ND	ND	2.45 + 0.21	ND	1.22 + 0.11	ND	1.01 + 0.10	4.00 + 0.34	2.84 + 0.21
Myrtenyl acetate	12.42	ND	ND	ND	ND	ND	ND	ND	1.37 + 0.11	ND
D-Limonene	12.6	ND	ND	ND	ND	7.91 + 0.79	ND	4.95 + 0.51	ND	0.40 + 0.04
Eucalyptol	12.72	62.16 + 6.11	11.39 + 1.11	19.49 + 1.87	46.28 + 4.25	22.70 + 2.14	37.36 + 3.60	9.35 + 0.87	18.36 + 1.75	0.40 + 0.03
1-Methyl-1-siabenzocyclobutene	12.92	ND	0.03 + 0.01	0.41 + 0.04	ND	ND	ND	1.54 + 0.11	ND	ND
*cis*-Sabinene	13.92	ND	ND	1.13 + 0.11	ND	0.67 + 0.06	ND	1.22 + 0.10	ND	ND
2,3-Decadiyne	14.57	ND	ND	0.29 + 0.02	0.28 + 0.02	ND	ND	0.55 + 0.02	1.08 + 0.12	0.28 + 0.02
*p*-Cymenene	14.66	ND	ND	ND	0.48 + 0.04	ND	ND	3.73 + 0.27	ND	0.22 + 0.02
Isopropenyltoluene	14.71	ND	ND	ND	ND	ND	ND	ND	ND	ND
1-Methyl-4-(methylidene)cyclohexane	14.78	ND	ND	ND	ND	ND	ND	ND	6.31 + 0.55	ND
6-Isopropyldiene-1-methyl-Bicyclo[3,1,0]hexane	14.98	ND	ND	ND	1.44 + 0.11	ND	ND	ND	3.84 + 0.37	ND
2-(Cyclopenta-1-enyl)-thiophene	15.49	0.08 + 0.01	ND	ND	ND	ND	ND	0.05 + 0.01	ND	0.55 + 0.05
1,3-Cyclohexadiene	15.84	ND	0.75 + 0.06	4.10 + 0.26	ND	ND	ND	ND	6.40 + 0.59	ND
2,4,6-Oxtatriene	16.23	ND	0.30 + 0.03	0.02 + 0.01	0.37 + 0.03	ND	ND	1.15 + 0.09	6.28 + 0.61	ND
l-Menthone	16.47	ND	ND	2.00 + 0.17	2.22 + 0.19	7.19 + 0.70	ND	0.96 + 0.09	1.71 + 0.11	ND
Menthol	16.57	4.30 + 0.33	ND	0.83 + 0.08	3.02 + 0.22	36.91 + 3.48	5.62 + 0.54	ND	2.11 + 0.25	11.29 + 1.08
2-Propyl-1,3-cyclohexadiene	16.66	ND	ND	0.01 + 0.01	ND	ND	ND	ND	0.23 + 0.02	ND
Acetic acid phenyl ester	16.8	ND	ND	0.02 + 0.01	ND	ND	ND	ND	ND	ND
Citronella	16.85	2.65 + 0.27	11.04 + 1.01	1.09 + 0.07	ND	ND	ND	ND	ND	1.84 + 0.14
α-Methyl cinnamic aldehyde	18.54	0.61 + 0.06	ND	0.32 + 0.02	ND	ND	ND	ND	ND	0.10 + 0.01
4-(5-Methyl-2-furanyl)-2-butanone	19.19	ND	ND	ND	0.70 + 0.58	2.41 + 0.19	ND	3.71 + 0.34	5.23 + 0.48	1.04 + 0.10
*p*-Methan-3-one	19.53	ND	ND	ND	ND	ND	ND	31.24 + 4.31	8.13 + 0.77	71.87 + 7.89
Thymol	20.63	ND	ND	1.09 + 0.11	ND	5.31 + 0.48	ND	5.41 + 0.49	ND	2.56 + 0.19
Ascaridole epoxide	20.75	ND	ND	ND	ND	0.02 + 0.01	0.40 + 0.04	3.36 + 0.31	ND	ND
Cubenol	21.38	1.14 + 0.12	0.92 + 0.08	ND	0.70 + 0.58	1.20 + 0.11	1.74 + 0.18	0.58 + 0.48	0.43 + 0.04	0.84 + 0.07
Santolinatriene	21.7	ND	ND	ND	0.96 + 0.80	0.80 + 0.07	ND	ND	ND	ND
α-Cubebene	22.02	0.17 + 0.01	0.72 + 0.06	0.01 + 0.01	0.46 + 0.38	0.02 + 0.01	0.56 + 0.05	ND	0.26 + 0.02	ND
(±)Camphorquinone	22.59	ND	0.42 + 0.04	51.61 + 4.79	ND	1.50 + 0.12	ND	3.84 + 0.37	0.20 + 0.02	1.93 + 0.20
Gemacrene D	22.73	ND	1.34 + 0.12	ND	0.88 + 0.07	0.50 + 0.04	ND	ND	0.47 + 0.04	ND
β-Yiangene	22.99	ND	ND	ND	ND	ND	ND	ND	ND	ND
α-Cedrene	23.07	ND	1.94 + 0.20	ND	ND	0.05 + 0.01	0.86 + 0.09	ND	ND	ND
β-Elemene	23.15	0.60 + 0.06	0.70 + 0.60	ND	0.82 + 0.08	0.04 + 0.01	0.51 + 0.06	ND	0.45 + 0.04	ND
*cis*-Muurola-3,5-diene	23.24	ND	0.62 + 0.05	ND	ND	0.39 + 0.03	0.22 + 0.03	ND	0.25 + 0.02	ND
2-Methylene-4,8,8-trimethyl-4-vinyl-bicyclo[5,2,0]nonane	23.54	ND	ND	0.01 + 0.01	1.44 + 0.11	0.29 + 0.02	0.22 + 0.02	ND	1.08 + 0.91	ND
α-Gurjunnene	23.6	ND	10.88 + 1.04	ND	ND	ND	0.34 + 0.03	ND	0.91 + 0.08	ND
Caryophyllene	23.86	5.50 + 0.40	8.61 + 0.73	1.01 + 0.14	5.47 + 0.49	1.05 + 0.12	2.42 + 0.25	0.48 + 0.04	9.81 + 0.76	1.36 + 0.10
Panasinsine	24.09	0.85 + 0.07	4.32 + 0.39	0.04 + 0.01	1.00 + 0.09	ND	ND	ND	0.57 + 0.49	ND
α-Bergamotene	24.24	ND	3.60 + 0.24	ND	0.02 + 0.01	0.30 + 0.01	ND	ND	ND	ND
Aromadendrene	24.37	0.17 + 0.01	1.16 + 0.10	ND	ND	0.07 + 0.01	2.24 + 0.20	ND	0.29 + 0.03	ND
α-Copaene	24.54	0.83 + 0.08	1.04 + 0.09	0.06 + 0.01	1.02 + 0.10	0.03 + 0.01	ND	0.75 + 0.07	0.19 + 0.02	ND
Guaia-1(10)-11-diene	24.62	ND	1.55 + 0.12	ND	ND	0.03 + 0.01	0.34 + 0.04	ND	1.47 + 0.13	ND
γ-Elemene	24.73	ND	0.62 + 0.04	ND	0.61 + 0.05	0.02 + 0.01	0.51 + 0.05	ND	1.02 + 0.11	0.02 + 0.01
γ-Muurolene	24.91	ND	0.79 + 0.08	0.01 + 0.01	ND	0.01 + 0.01	0.27 + 0.03	ND	0.39 + 0.04	ND
(+)-epi-Bicyclo sesquiphellandrene	24.97	1.30 + 0.11	1.18 + 0.20	ND	1.28 + 0.11	0.61 + 0.06	0.37 + 0.04	ND	ND	0.04 + 0.01
Isoledene	25.08	0.86 + 0.07	1.25 + 0.20	ND	ND	ND	0.22 + 0.03	1.09 + 0.10	ND	ND
δ-Cadinene	25.22	ND	ND	ND	0.01 + 0.01	0.02 + 0.01	0.18 + 0.02	ND	ND	0.01 + 0.01
β-Copaene	25.29	ND	ND	ND	0.04 + 0.01	0.26 + 0.02	0.13 + 0.01	ND	0.38 + 0.02	0.02 + 0.01
α-Muurolene	25.37	ND	1.21 + 0.19	ND	0.02 + 0.01	ND	0.19 + 0.02	ND	0.28 + 0.03	0.01 + 0.01
α-Curcumene	25.45	0.18 + 0.01	2.33 + 0.20	0.23 + 0.02	ND	0.14 + 0.01	0.05 + 0.01	ND	ND	ND
Isosativene	25.61	ND	0.98 + 0.08	0.15 + 0.01	ND	ND	0.35 + 0.03	ND	ND	ND
(+)-Ledene	25.75	0.43 + 0.04	2.28 + 0.21	0.13 + 0.01	0.36 + 0.03	ND	1.84 + 0.19	0.21 + 0.02	0.98 + 0.08	ND
(−)-Aristolene	25.86	0.14 + 0.01	2.50 + 0.22	0.37 + 0.03	0.25 + 0.02	0.01 + 0.01	ND	ND	0.77 + 0.08	ND
(+)-Cyclosativene	26.21	0.42 + 0.03	1.91 + 0.13	0.04 + 0.01	ND	0.28 + 0.02	2.00 + 0.20	ND	0.47 + 0.50	0.02 + 0.01
(−)-Calamenene	26.43	2.44 + 0.19	12.17 + 1.22	0.07 + 0.01	3.34 + 0.21	0.33 + 0.03	9.22 + 0.91	ND	5.09 + 0.43	0.92 + 0.08
β-Euaiene	26.77	0.17 + 0.01	1.04 + 0.11	ND	0.01 + 0.01	0.04 + 0.01	ND	ND	0.33 + 0.03	ND
α-Calacrene	26.93	ND	1.03 + 0.09	ND	0.11 + 0.01	0.02 + 0.01	ND	ND	0.33 + 0.05	ND
Epiglobulol	28.13	ND	0.99 + 0.08	ND	ND	0.03 + 0.01	ND	ND	0.49 + 0.03	ND
Mansonone C	28.78	ND	ND	ND	ND	ND	ND	ND	0.54 + 0.04	0.01 + 0.01
Di-*tert*-butyl-4-sec-butylphenol	29.02	ND	ND	0.10 + 0.01	ND	0.01 + 0.01	ND	2.99 + 0.19	0.25 + 0.02	0.07 + 0.01
Decanoic acid octyl ester	29.14	ND	ND	ND	ND	ND	ND	1.14 + 0.10	ND	ND
Sinerol	29.86	1.44 + 0.09	0.04 + 0.01	ND	ND	0.07 + 0.01	ND	0.97 + 0.08	0.67 + 0.05	0.19 + 0.01
Azulol	30.02	ND	0.09 + 0.01	ND	ND	ND	ND	ND	0.55 + 0.04	ND
3,5-*Bis*(*tert*-butyl)-4-hydroxy-propiohenon	30.23	ND	0.85 + 0.08	ND	ND	ND	ND	0.49 + 0.04	ND	ND
(6-*tert*-Butyl-1,1-dimethyl-2,3-hydro-1H-inda-4-yl) acetic acid	30.89	ND	ND	ND	ND	ND	ND	1.23 + 0.11	ND	0.02 + 0.01
5α-androstane	32.53	ND	ND	ND	ND	ND	ND	1.12 + 0.11	ND	ND
Total	99.67 ± 9.24	98.27 ± 9.61	94.95 ± 9.41	98.54 ± 9.32	97.42 ± 8.83	99.70 ± 10.93	97.02 ± 9.54	99.70 ± 9.92	97.84 ± 10.55	99.81 ± 10.42

*RT* retention time (min), *ND* not detected, *AP* apple mint, *CC* chocolate mint, *ED* eau de cologne mint, *HS* horsemint, *PA* pineapple mint, *PP* peppermint, *PR* pennyroyal mint, *SP* spearmint, *St* streptomicine, *WT* water mint

^a^As mean ± SD (standard deviation) of triplicate experiments

### Antimicrobial screening

*Mentha* species are one of the ornamental flowering plant found in South Korea. The application of this plant was reported by demonstrating its in vitro antibacterial and antioxidant properties. First, we screened the different crude organic extracts of nine *Mentha* species showed that the ethanol extract having more activity against six pathogenic bacteria. Disc diffusion method revealed that the ethanol extract produce more activity than other organic solvents compared to other extracts (data not shown). Nine mint species of ethanol extracts having significant activity against *S. haemolyticus* and then followed *E.coli* (KF 918342), *C. sakazakii* (ATCC 29544), *A. salmonicida* (KACC 15136), *E.coli* (ATCC 35150) and *A. hydrophila* (KCTC 12487). Among these mint species, chocolate mint having more activity compared to other species followed by the horsemint, pennyroyal mint, eau de cologne mint, peppermint, apple mint, water mint, spearmint, and pineapple mint. These results were showed in Table 2.

**Table 2:** Antimicrobial activity from the extracts of *Mentha* species

	*E. coli* (KF 918342)	*S. haemolyticus*	*A. hydrophila*	*E. coli* (ATCC 35150)	*C. sakazakii*	*A. salmonicida*
PP	19.00 ± 0.00	15.67 ± 0.58	15.00 ± 1.00	20.00 ± 0.00	15.33 ± 1.53	13.67 ± 0.58
WT	16.00 ± 0.00	14.67 ± 1.53	14.33 ± 2.89	10.00 ± 0.00	11.67 ± 2.08	9.67 ± 1.53
AP	13.67 ± 0.58	15.00 ± 0.00	15.67 ± 2.08	13.00 ± 0.00	15.00 ± 2.00	11.33 ± 1.53
SP	9.00 ± 1.00	20.33 ± 2.08	20.33 ± 0.58	13.00 ± 1.00	16.00 ± 1.73	15.67 ± 1.15
CC	20.67 ± 1.15	22.00 ± 1.00	19.33 ± 0.58	22.00 ± 0.00	17.67 ± 0.58	16.67 ± 1.53
PA	15.00 ± 1.00	8.67 ± 0.58	12.00 ± 0.00	14.00 ± 1.00	13.33 ± 0.58	8.33 ± 1.53
HS	20.33 ± 1.15	21.33 ± 1.15	18.67 ± 1.53	20.00 ± 0.00	16.67 ± 3.06	15.67 ± 1.15
ED	19.00 ± 0.00	21.33 ± 0.58	21.00 ± 0.00	20.00 ± 1.00	15.33 ± 1.15	15.00 ± 1.00
PR	20.33 ± 1.15	20.67 ± 1.15	17.67 ± 0.58	22.33 ± 0.58	18.00 ± 1.00	19.67 ± 0.58
St	28.00 ± 0.00	25.67 ± 0.58	26.67 ± 0.58	27.33 ± 0.58	26.00 ± 0.00	28.00 ± 0.00

Zone of inhibition (mm) of ethanol extract from different species of *Mentha. AP* apple mint, *CC* chocolate mint, *ED* eau de cologne mint, *HS* horsemint, *PA* pineapple mint, *PP* peppermint, *PR* pennyroyal mint, *SP* spearmint, *St* streptomicine, *WT* water mint

## Discussion

Various factors including physiological variations, environmental conditions, geographic variations, genetic factors and evolution, political/social conditions, amount of plant material/space, and manual labor needs determine chemical variability and yield, viz. the volatiles and those occurring in essential oils, for each species (Figueiredo et al. 2008). Likewise, chemotype of the plants, cultivation and processing methods also cause differences in chemical composition (Pavela 2009). From our results, differences in each chemical profile were observed from nine species of *Mentha*. We found that eucalyptol (62.16 %) is dominant and 4-terpineryl acetate (6.17 %), caryophyllene (5.50 %), menthol (4.30 %) are accumulated slightly in peppermint. This is in disagreement with the results of Zhenliang Sun et al. (2014). They evaluated that menthol (30.69 %), menthone (14.51 %), and menthy acetate (12.86 %) are present dominantly in peppermint. Previous study indicated that methofuran (51.27 %) limonene (12.06 %), and isomenthone (8.11 %) were contained largely in *M. aquatica*; water mint (Zamfirache et al. 2010). However, we found that (−)-calamenene (12.17 %), eucalyptol (11.39 %), and citronellal (11.04 %) are detected at significant concentration in water mint. The most abundant components in horsemint were *p*-methan-3-one (31.24 %), eucalyptol (9.35 %), and thymol (5.41 %). These results showed differences from the findings of Koliopoulos et al. (2010). They have demonstrated that *M. longifolia*; horsemint has piperitone oxide (33.4 %), 1,8-cineole 24.5 %, and trans-piperitone epoxide (17.4 %) from central Greece and carvone (54.7 %), limonene (20.0 %), β-pinene and piperitone (5.0 %, respectively) from East-Southern Greece as the major volatiles. The volatile extracts from pennyroyal mint were found to be rich in *p*-menthan-3-one (71.87 %), followed by menthol (11.29 %) in this study. Similarly, the major groups of components were oxygenated monoterpenes (82.8–85.2 %) in pennyroyal mint (Díaz-Maroto et al. 2007). Earlier analysis has shown that the greatest level of menthol was detected in chocolate mint and eau de cologne mint yielded the highest essential oil content. Also, it has been reported that *M. suavelons* such as pineapple mint shows lower amount of essential oil than other varieties (Gracindo et al. 2006). These findings are corresponded well. In *M. spicata*, carvone (49.5 %) and menthone (21.9 %) were identified as main volatiles among 27 components (Soković et al. 2009).

The ethanol extracts obtained from nine *Mentha* species were screened antibacterial activity. The pepper mint oil having antibacterial against *E. coli* and *S. aureus* has been investigated by Rasooli et al. (2007). Although different organic extracts of antibacterial activity against the *E. coli* and *S. aureus* bacterial strains were reported by Priya et al. (2007). The plant oil and extracts have been sensitive effects proved against those Gram-positive bacteria compared to Gram-negative bacteria (Cosentino et al. 1999; Karaman et al. 2003; Şahin et al. 2003). In recent years plants used as medicines traditionally, increasing antibiotic resistance against pathogenic bacteria and undesirable side effects of antibiotics indicated the *Mentha* essential oils have been used as antibiotics or alternatives for the treatment of various infectious diseases. Many researchers investigated various plant extracts and essential oils have been used as topical antiseptics and reported to possess antimicrobial properties. There is a requirement to explore scientifically, novel antimicrobial compounds produced from the plant oils and extracts, which have been used in traditional medicines as important sources (Mitscher et al. 1987).

Antibacterial and antifungal activities of *Mentha* species have been investigated in the previous studies (Karaman et al. 2003; Sahin et al. 2003; Kitic et al. 2002). The mint essential oils also revealed the antibacterial activity were previously reported against *S. aureus*, *E. coli* and *Klebsiella* spp. (Jeyakumar et al. 2011; Chauhan and Agarwal 2013; Sujana et al. 2013). In this study, the ethanol extract of chocolate mint showed highest activity against *S. haemolyticus* and also having significant inhibition activity against *E.coli* (KF 918342), *C. sakazakii* (ATCC 29544), *A. salmonicida* (KACC 15136), *E.coli* (ATCC 35150) and *A. hydrophila* (KCTC 12487), whereas thelowest activity showed in pineapple mint.

Studies have shown the effects of plant volatiles and their component on bacterial properties (Lis-Balchin and Deans 1997). These activities are thought to be involved in composition, structural form, and functional family or potential interaction between constituent components of plant volatiles (Dorman and Deans 2000). Among components, terpenes have known as antimicrobial agents against various microorganisms, both Gram-positive and Gram-negative bacteria and fungi (Cowan 1999). Our findings have shown that *Mentha* species are characterized by a high content of monoterpenes like eucalyptol, menthol, and so on. In addition, all plants indicated high levels of inhibition against bacteria strains. Similarly, the essential oils from oregano and thyme which indicate high levels of monoterpenes such as hydrocarbons and oxygenated compounds have well known for antimicrobial activity (Baratta et al. 1998; Azaz et al. 2004). Several monoterpenes have investigated the antibacterial properties in many researches. For example, Trombetta et al. have demonstrated the antimicrobial action of thymol and menthol (Trombetta et al. 2005). Eugenol also inhibits viability of thirty *Helicobacter pylori* strains (Wang et al. 2012). Chemical structures of monoterpenes and target organisms have effect on their various pharmacological properties (Koziol et al. 2014). In some studies, total volatile represents better antibacterial activity than the specific components. It seems that minor components would lead to synergistic effect on the activity (Gill et al. 2002; Mourey and Canillac 2002).

## Conclusions

Taken together, the present study characterized and identified 77 volatile compounds from *Mentha* species in total. Interestingly, monoterpenoids including eucalyptol, menthol, and *p*-methan-3-one were largely accumulated and detected as main constituents in these plants. Besides, our results demonstrated that the extracts from chocolate mint, horse mint, and pennyroyal mint which principally contains menthol and *p*-methan-3-one lead to high antimicrobial activity. We suggest that these *Mentha* species which indicated high antimicrobial activity are have potential in diverse commercial industries such as pharmaceutical, food, and cosmetic. Although the antibacterial properties and compositions of volatile have been investigated, the correlations are still unclear in detail. Furthermore, we would focus on characterization of the mode of action and synergism between components from volatiles.

## Methods

### Plant materials

**Fig. 1:**
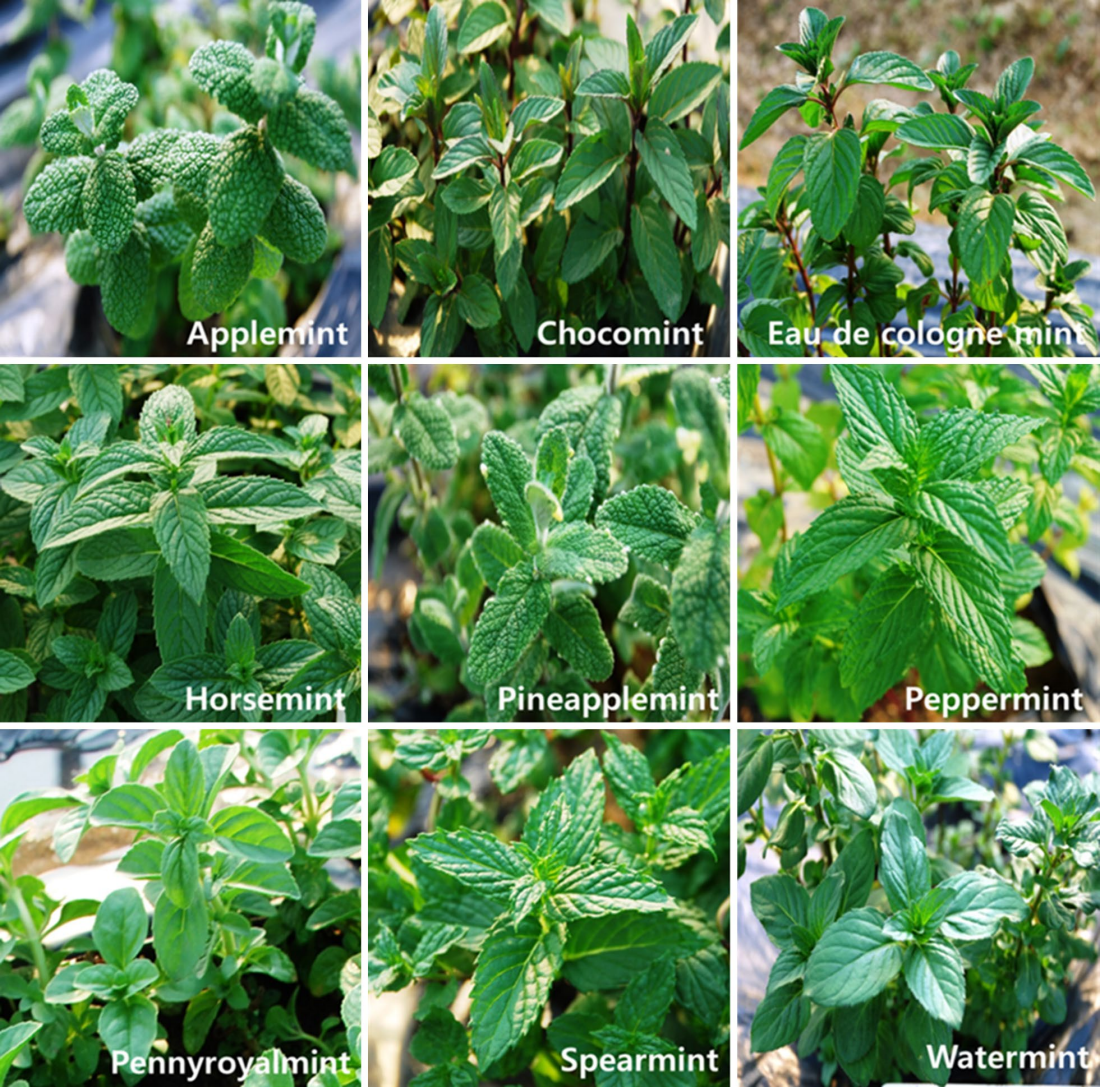
Photographs of nine *Mentha* spp Pictures were taken by Y. J. Park

The young seedlings of nine *Mentha* species including *M. piperita*; peppermint, *M. pulegium*; pennyroyal mint, *M. spicata*; spearmint, *M. longifolia*; horse mint, *M. aquatica*; water mint, *M. suaveolens*; apple mint, *M. suaveolens* ‘Variegata’; pineapple mint, *M. piperita* f. citrate; choco mint, and *M. piperita* var. citrate; eae de cologne mint were purchased from Seed Mall Co. (Seoul, Korea) (Fig. 1). Each *Mentha* plants were established in a greenhouse at the experimental farm of Chungnam National University (Daejeon, Korea). After 4 months, aerial parts of these plants were harvested.

### Analysis of GC and GC-mass spectrometry

Gas chromatography (GC)- Mass spectrometry (MS) analysis was carried on a 7820A GC/5977E MSD (Agilent, USA) fitted with an HP-5 (30 m × 0.25 mm ID, film thickness 0.25 µm) fused-silica capillary column (Agilent, USA). The carrier gas was helium at flow rate of 1.0 ml/min. The mass spectra were obtained with an ionization voltage 70 eV, trap current 250 μA, and ion source temperature of 200 °C. The conditions of programmed oven temperature were similar to those described for GC. Samples were injected using the splitless mode. The column temperature was maintained at 35 °C for 2 min and programmed as follows: increase rate 5 °C/min to 250 °C and finally hold for 10 min at 250 °C.

Each *Mentha* plants (2.0 g) were put into a 15 mL thermostated vial and the SPME fiber was introduced for 12 h into the thermostated vial (RT) with a rubber septum containing 2.0 g of the three fresh aerial parts of each *Mentha* species during the SPME extraction procedure. A 1 cm long 50/30 µm polydimethylsiloxane/divinylbenzene/carboxen-coated fiber was utilized for analysis. The fiber was adjusted in a GC injection port at for 1 min prior to use. The absorbed component was injected into a GC by desorption at 250 °C for 2 min in the injector (splitless mode). SPME procedure was carried out three times and the results were presented as the mean ± standard deviation.

### Preparation of the extracts for antibacterial activities

Dried samples of nine *Mentha* species were extracted with different solvents such as ethanol, methanol, hexane, diethyl ether and ethyl acetate. 10 g of powdered sample was soaked in 50 mL of different solvents for 1 day after that the extract filtered using filter paper. Then filtrate was evaporated using rotary vacuum evaporator and powdered sample were stored for 4 °C for further experiments (Santhosh et al. 2016).

### Bacterial strains and cultivation

Bacterial strains including *E.coli* (KF 918342), *Stephylococcus haemolyticus, Aeromona shydrophila* (KCTC 12487), *E.coli* (ATCC 35150), *Cronobacter sakazakii*(ATCC 29544), *Aeromonas salmonicida* (KACC 15136) were used for experiment. These six strains were collected from medicine department of Chungnam National University. 50 ml of LB broth was prepared in 250 ml conical flask and the bacterial strains were grown in this medium at 37 °C on an orbital shaker. The culture flasks were inoculated at 0.1 OD600 nm with freshly prepared LB medium under same culture conditions (Rejiniemon et al. 2015). The mid log phase bacterial cultures were used for the antibacterial studies. *S. haemolyticus* are only the Gram positive bacteria used in this study all other Gram negative bacteria.

### Disk diffusion method

0.1 OD of overnight different bacterial cultures was swabbed on the 25 ml LB agar plates. Then the whatman disk was placed on the plates. About 30 ul of different solvent extract of different mint species were add on that whatman disc and incubate for overnight at 37 °C. Ethanol, methanol, hexane, diethyl ether and ethyl acetate used as a control and streptomycine was used as a standard (Balachandran et al. 2015).

### Statistical analysis

All analysis was performed using three biological replicates. Moreover, general statistical analyses for standard deviations were conducted using the database from experimental processes by Microsoft Corporation, Seattle, WA, USA).

## Abbreviations

AP: apple mint
CC: choco mint
CDP-ME: 4-(cytidine 5′-diphospho)2-*C*-methyl-D-erythritol
CDP-ME2P: 4(cytidine 5′-diphospho)-2-*C*-methyl-D-erythritol phosphate
CNS: central nervous system
DMAPP: dimethylallyl diphosphate
ED: eau de cologne mint
FPP: farnesyl diphosphate
GA-3P: glyceraldehyde-3-phosphate
GC: gas chromatography
GPP: geranyl diphosphate
HMBPP: (E)-4-hydroxy-3-methylbut-2-enyl diphosphate
ME-2,4cPP: 2-*C*-mehtyl-D-erythritol 2:4-cyclodiphosphate
HS: horsemint
IPP: isopentenyl diphosphate
MVA: mevalonic acid
MEP: methylerythritol phosphate
AcAc-CoA: acetoacetyl-CoA
PA: pineapple mint
PP: peppermint
PR: pennyroyal mint
SP: spearmint
St: streptomicine
WT: water mint
